# Assessing uncertainty in model parameters based on sparse and noisy experimental data

**DOI:** 10.3389/fphys.2014.00128

**Published:** 2014-04-04

**Authors:** Noriko Hiroi, Maciej Swat, Akira Funahashi

**Affiliations:** ^1^Systems Biology Laboratory, Department of Bioscience and Informatics, Keio UniversityYokohama, Japan; ^2^European Molecular Biology Laboratory, European Bioinformatics Institute, Hermjakob Team: ProteomicsCambridgeshire, UK

**Keywords:** parametric identification, generalized least squares, sensitivity analysis, fisher information matrix, bifurcation analysis

## Abstract

To perform parametric identification of mathematical models of biological events, experimental data are rare to be sufficient to estimate target behaviors produced by complex non-linear systems. We performed parameter fitting to a cell cycle model with experimental data as an *in silico* experiment. We calibrated model parameters with the generalized least squares method with randomized initial values and checked local and global sensitivity of the model. Sensitivity analyses showed that parameter optimization induced less sensitivity except for those related to the metabolism of the transcription factors c-Myc and E2F, which are required to overcome a restriction point (R-point). We performed bifurcation analyses with the optimized parameters and found the bimodality was lost. This result suggests that accumulation of c-Myc and E2F induced dysfunction of R-point. We performed a second parameter optimization based on the results of sensitivity analyses and incorporating additional derived from recent *in vivo* data. This optimization returned the bimodal characteristics of the model with a narrower range of hysteresis than the original. This result suggests that the optimized model can more easily go through R-point and come back to the gap phase after once having overcome it. Two parameter space analyses showed metabolism of c-Myc is transformed as it can allow cell bimodal behavior with weak stimuli of growth factors. This result is compatible with the character of the cell line used in our experiments. At the same time, Rb, an inhibitor of E2F, can allow cell bimodal behavior with only a limited range of stimuli when it is activated, but with a wider range of stimuli when it is inactive. These results provide two insights; biologically, the two transcription factors play an essential role in malignant cells to overcome R-point with weaker growth factor stimuli, and theoretically, sparse time-course data can be used to change a model to a biologically expected state.

## Introduction

Parametric identification is a significant process of model building. The identification problem concerns the possibility of drawing inferences from observed samples to an underlying theoretical structure. The basic results for linear simultaneous equation systems under linear parameter constraints were found in 1950, and extensions to non-linear systems and non-linear constraints were made by Fisher (1961) and others.

There exist some steps of parametric identification: (1) checking structural identifiability, to clarify practical difficulties such as multimodality and lack of practical identifiability; (2) analysing sensitivity and ranking parameters; (3) model calibration including problem formulation, numerical solution, and global optimization methods of parameters; and based on this knowledge, performing (4) optimal experimental design.

These processes are performed to explain observed biological phenomena, or to fill gaps between the molecular level and larger patterns. Meanwhile, we may identify the key mechanisms of a system in a model, which can allow us to predict missing components, concepts, or unobserved phenomena, and serve as a guide for further experiments.

During each division cycle, cells need to duplicate their genomes and distribute the two copies equally to the two daughter cells. The processes of DNA-duplication (S-phase) and cell division (mitosis) are separated by two gap phases (G1 and G2). During these phases, several mechanisms operate to prevent cells from continuing the cell cycle under inappropriate conditions. Normal cells can interrupt the cell cycle in the gap phases through growth inhibitory mechanisms that activate the retinoblastoma proteins (Rb) or p53 transcription factors. In cancer cells, these growth inhibitory pathways are often disrupted, leading to unscheduled proliferation (Hanahan and Weinberg, 2000).

We used Yao's 2008 model (Yao et al., 2008), which is consistent with experimental data exhibiting bimodality. The model represents the underlying mechanisms of a restriction point (R-point), which is the critical event for a mammalian cell to commit to proliferation independently from extracellular growth stimuli.

Normal cells respond to extracellular growth factors. Their absence arrests the cell cycle in the G1 phase. However, growth factors are required only until a few hours prior to the initiation of S-phase. This moment in G1 was first described in 1974 by Pardee (1974) and is named the R-point. It was clarified later that cells that pass the R-point can progress to S-phase independently of mitogens (Sherr and Roberts, 2004). Importantly, Pardee found that the R-point was defective in cancer cell lines. In addition, cancer cells were much more resistant to the inhibition of protein synthesis, which is supposed to be required for the R-point, suggesting that the required R-point factors are either stabilized in cancer cells or not necessary to progress the cell cycle (Campisi et al., 1982). An example of their findings is when the Rb protein has its activity inhibited, and the machinery of the R-point is disrupted and the cell lines are transformed into malignant lines.

This model correctly reconstructs the most fundamental behavior of the molecular network system of the mammalian cell cycle, such as bimodality, by its structure. The molecular mechanism, which this model represents, is also significant to control the switching among different physiological cellular states: from normal cell proliferation to malignant, or differentiation and cell death. These switching mechanisms between normal proliferation and other states are the key to tumourigenesis, the variation in leukocyte production, and so on. The missing property of this model is that it has never been fitted to a time-course data of molecules. There exist other models that represent cell cycle mechanisms; however, many of them have not yet been tested with high resolution experimental data to follow the dynamics of the system. This is a difficulty when using mathematical models, even if they have good potential to predict important insights.

The model calibration problem consists of finding a model to minimize the distance among model predictions and the experimental data. There exist several strategies for model calibration. One is the maximum likelihood. In this analysis, a probabilistic distribution in the noise is considered but without considering any uncertainty in the parameters. Another is Bayesian estimation, which introduces information about a prior probabilistic distribution of the parameters and noise.

We applied generalized least squares for our parameter optimization, which requires almost no prior information (Balsa-Canto et al., 2008). Prior to and after optimization, we performed both local sensitivity analysis (LSA) and global sensitivity analysis (GSA) (Rodriguez-Fernandez and Banga, 2010). LSA is usually performed to measure how sensitive the model is to small changes in the original parameter values that are first given. On the other hand, GSA is performed to measure how sensitive the model is to changes in the parameters over the full range of plausible values. The objective of performing the sensitivity analyses was to rank the parameters in order of importance for observation, then use the rank to assist in fixing parameters to improve practical identifiability.

In order to find necessary additional information through experiments, analysing the parameter sensitivity and checking the global ranking and identifiability are needed (Balsa-Canto and Banga, 2011). We used these results to design several rounds of parameter optimization. The objective of the ranking was to assess the importance of individual parameters. Several criteria have been suggested to locally rank parameters (Balsa-Canto and Banga, 2011). Relative local parametric sensitivities are computed for a number of *n*_Ihs_ samples using the Latin Hypercube Sampling approach within parameter bounds to generalize it to a global rank (Balsa-Canto and Banga, 2011).

We performed bifurcation analysis to understand how the parameter calibration affected the behavior of the model (Ermentrout, 2002). Many numerical models, when applied to real biological systems, involve non-linearities that make possible the model's chaotic behavior and oscillation. At the same time, many models are difficult to solve analytically because of their complex structure. Numerical solutions have an advantage in such cases in that they can be used to perform further analyses with those models. The cell cycle model we chose shows oscillation as one of the characteristics of this model. Bifurcation analysis allowed us to test how the characteristics of the systems depend on the parameters. Two-parameter curves show us a range of parameters that may produce multiple states.

Here, we describe all the above investigation results and discuss the potential of parameter fitting to a sparse dataset to improve model behavior when representing physiological conditions. Finally, we discuss how to make further improvements with additional experiments and simulations.

## Methodology

### Model and data

**Figure 1:**
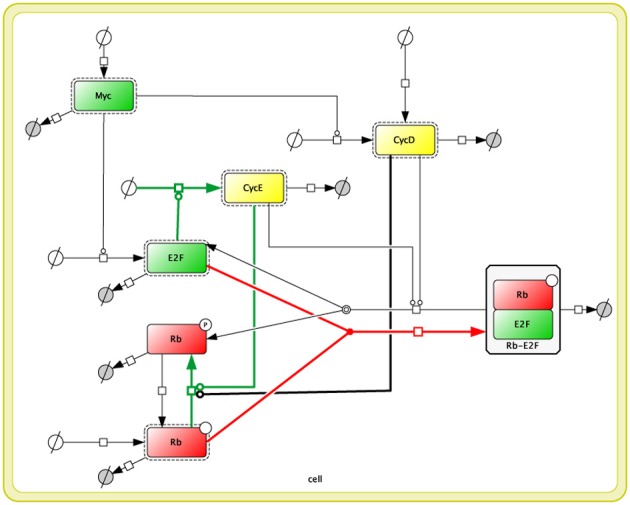
**Reconstructed diagram of Yao's 2008 model (Yao et al., 2008) in Cell Designer (Funahashi et al., 2008)**. Each square indicates protein, and rectangles with dotted lines indicate activated forms of those proteins. The whole the diagram is included inside a compartment, which represents a cell, with double yellow lines. White circles with a crossing line indicate a reactant source, and gray circles with a crossing line indicate waste. All edges correspond to the fluxes from a reaction species to the others.

The model we used for our analyses was originally published by Yao et al. (2008) and was analyzed following the procedures listed below. A diagram of the reconstructed model is shown in Figure 1, and the differential equation set is shown in the Appendix. The experimental data, which we used for the parameter fitting, were produced as described in the Experimental Methods.

### Model reconstruction

We reproduced Yao's 2008 Model with Cell Designer (Funahashi et al., 2008). The Yao 2008 model is in Biomodels.net (Chelliah et al., 2013) (no.318). We imported the Systems Biology Markup Language (SBML) (Hucka et al., 2004) file (BIOMD0000000318.xml) to CellDesigner, and then recreated it as a reaction network. All the kinetic laws, parameters, and annotations (RDF) from biomodels.net were kept in the model.

We modified the reaction network so as to be close to that described in Yao's study (Yao et al., 2008). The model consists of 7 ODEs (Appendix), thus there are 7 species (proteins) in our version of the reaction network (Figure 1). Nevertheless, there are only 5 proteins in Yao's network as shown in Yao's Figure 1 (Yao et al., 2008). We assume that this happened because they had omitted two of the reaction species in their figure to focus on the activation-inhibition process of the network to simplify the diagram; as a result, inactive proteins are not shown in their figure. We included these inactive reaction species to rebuild their model correctly.

In our reaction network, the above 5 proteins in Yao's Figure 1 (Myc, E2F, Rb, CycD, and CycE) are shown as “Active” proteins (which have dashed rectangles around the proteins), and the other 2 “Inactive” proteins (phosphorylated Rb and Rb-E2F complex) are required to express the original mathematical model (to be 7 ODEs). Highlighted reactions (colored in green, red, and black) in the model are mapped to the reactions in Yao's original figure. We confirmed that our modified model generates the same simulation results as the original BIOMD0000000318.xml.

### Analysis methods

We used the Matlab toolbox Advanced Model Identification using Global Optimization (AMIGO) (Balsa-Canto and Banga, 2011), which includes options for local and global sensitivity analyses, local and global ranking of parameters, parameter estimation, and Fisher Information Matrix evaluation. XPPAUT (Ermentrout, 2002) was used for the basic simulation and bifurcation analysis of the model. In the following sections, we briefly describe each analysis.

#### Parameter optimization

We performed model calibration by generalized least squares because the method does not require any prior information of the model. The generalized least squares is described as:

$$\text{J}(\theta )\;=\sum_{\varepsilon \;=\;1}^{{\text{n}}_{\varepsilon }}\sum_{O\;=\;1}^{{\text{n}}_{\text{O}}{}^{\varepsilon }}{\left({\text{y}}^{\varepsilon ,\;O}(\theta )-y{\text{m}}^{\varepsilon ,\text{O}}\right)}^{\text{T}}{\text{Q}}^{\varepsilon ,\;O}({\text{y}}^{\varepsilon ,\;O}(\theta )-y{\text{m}}^{\varepsilon ,O})$$

where Q is the quadratic cost function. In our case, we used “standard least squares” with constant variance. Briefly, this is encoded as

inputs.PEsol.PEcost_type=“lsq”; % “lsq” (weighted least squares default) | “llk” (log likelihood) | “user_PEcost” inputs.PEsol.llk_type=“homo”; % to be defined for llk function, “homo” | “homo_var” | “hetero”

where “lsq” indicates Weighted Least Squares Funtion. For the cases where no information about the experimental error is available, “homo” is given homoscedastic noise with known constant variance.

θ, which gives minimum *J*(θ), is the least square estimator. This method can provide the best estimate for a linear model. Q^ε, *O*^ is a non-negative definite symmetric weighting matrix. The weighting coefficients ω^ε, *O*^_S_*S* = 1,…,n^ε, *O*^_S_ located in the diagonal of the matrix are positive or zero and fixed *a priori*. Basically, if ω_S_ = 1, it means to assign the same level of importance to all data; if ω_s_ = 0, it means a datum is eliminated because it is deemed not relevant; if ω_S_ = max(*y*m^ε, *O*^)^2^, the square of the maximum experimental data for the observable O and the experiment ε reduces the effect of having observations of different orders of magnitude. We used objective value in order to estimate if the parameter optimization improved fitting of the model to our experimental data. It is also mentioned frequently as residual standard error, and known if the value is exactly 0 then the model fits the data perfectly.

#### Local sensitivity analysis (LSA)

Local (Relative) Sensitivity Analysis (LSA) was performed with AMIGO for the case of (a), with original parameter settings of Yao's model, and (b), optimized parameters with our experimental data, to rank the parameters in order of importance for the observable variables.

#### Rank parameters based on LSA

The parameters were ordered according to the value of S^ε, *O*^_*p*_. We used the R programming language to produce the graphs of LSA results (R Development Core Team, 2008).

#### Global sensitivity analysis (GSA)

Global Sensitivity Analysis (GSA) was performed to measure how sensitive the observables are to changes in the parameters over the full range of plausible values: (a), with default values of Yao's original model, and (b), with optimized parameters based on experiments. We assessed the importance of individual parameters and also ranked parameters based on the results of GSA, the criteria of which were originally suggested by Brun et al. (2001), but were extended to the formula shown below by Balsa-Canto et al. (Balsa-Canto and Banga, 2011). The result of parameter ranking based on GSA is indicated by the order of decreasing m_sqr_, which is best suited to serve as a ranking criterion (Balsa-Canto and Banga, 2011).

m_sqr_is defined as:

$$\delta^{\text{msqr}}{}_{\text{p}}=\frac{1}{{\text{n}}_{\text{Ihs}}{\text{n}}_{\varepsilon }{\text{n}}_{\text{O}}{\text{n}}_{\text{S}}}\sqrt{\sum_{\text{Ihs=1}}^{{\text{n}}_{\text{Ihs}}}\sum_{\varepsilon =1}^{{\text{n}}_{\varepsilon }}\sum_{O=1}^{{\text{n}}_{\text{O}}}\sum_{\text{S=1}}^{{\text{n}}_{\text{S}}}{\left({\text{S}}^{\varepsilon ,\text{O}}{}_{\text{p}}({\text{t}}^{\varepsilon ,\text{O}}{}_{\text{p}})\right)}^{2}}$$

We used the R programming language to produce the graphs of GSA results (R Development Core Team, 2008).

#### Bifurcation analysis

We performed Bifurcation analysis of Yao's model with (a) default and (b) optimized parameters by XPPAUT. Bifurcation analysis was based on the parametric dependence of dynamic systems encoded as differential equations. This approach is called the continuation method. Its name is derived from the fact that the number and type of steady states can vary as a function of one or more parameters. Typically, one starts with a stable steady state and then varies a particular parameter in very small increments and calculates the type of the steady state at the next point of parameter space. The parameter we used here was the stimulus, S. For the 2-dimentional bifurcations plots, we scanned S vs. the number of other parameters. We let XPPAUT scan the region around their default or their optimized values starting at a low stable steady state. We defined the range from 0.1 to 10 times their starting values for each parameter to test, and between 0.0 and 1.5–2.5 for the stimulus, S.

### Experimental methods

#### Cell culture and synchronization

3Y1 rat embryonic fibroblasts were cultured in 5% CO_2_ at 37°C in Dulbecco's modified Eagle's medium (DMEM) supplemented with 10% fetal calf serum (FCS) (Hiroi et al., 2006). Cell synchronization was performed by the thymidine double block (Hiroi et al., 1999). Exponentially dividing cells were incubated at 37 ±°C for 18 h in medium containing 0.56 mM 20-deoxythymidine. Then the cells were washed with fresh DMEM-10% FCS without 20-deoxythymidine and then recultured for 15 h in drug-free medium. The cells were synchronized at the next G1/S boundary by incubating them for a further 15 h in medium containing 0.56 mM 20-deoxythymidine. After the removal of the second thymidine-block, cells were harvested at the indicated times and subjected to flow cytometry.

#### DNA flow cytometry

DNA content was determined by flow cytometry. 5 × 10^5^ cells were washed once in phosphate buffered saline (PBS) and fixed in 70% ethanol for 30 min on ice. The cells were centrifuged at 400 × *g* for 5 min, and the pellet was incubated at 37°C for 20 min in 500 μl of PBS containing 0.1 mg/ml RNase A. The cells were then pelletted and stained with 100 μl of 25 μg/ml propidium iodide in PBS. Finally, the stained cells were suspended in 0.1% BSA/PBS and analyzed using a flow cytometer (Beckman-Coulter). The data were acquired and analyzed by the provided computer program (Beckman-Coulter, WinCycle). A sequence of single-parameter DNA histograms was analyzed to determine the proportions of cells in each phase.

#### Western blot detection

Western blot analysis was performed as described (Hiroi et al., 2002). In preparation for western blotting, 5 × 10^5^ cells were lysed in 100 μl of radioimmunoprecipitation (RIPA) buffer (150 mM NaCl, 1% NP-40, 0.1% sodium dodecyl sulfate (SDS), 50 mM Tris-HCl (pH 7.5), 0.1 mM Na-orthovanadate, 0.1 mM NaF, 1 mM dithiothreitol (DTT), 1 mM phenylmethylsulfonyl fluoride, 1 μg/ml pepstatin, 1 μg/ml leupeptin, and 1 μg/ml aprotinin). After a 10 min incubation on ice, lysed cells were centrifuged at 20 000 × *g* for 10 min at 4°C. After adjustment of the protein concentration, the supernatants were used for western blotting. The proteins or control peptide for each target protein in SDS loading buffer (2% SDS, 10% glycerol, 60 mM Tris-HCl, 100 mM DTT, and 0.001% bromophenol blue) were boiled for 5 min, separated by SDS-polyacrylamide gel electrophoresis (16% polyacrylamide gels), and blotted onto Immobilon-P^*SQ*^ membranes (Merck Millipore, Billerica, MA). Sample transfer was confirmed with gel staining (coomassie brilliant blue; CBB) and a secondary-layered backup membrane. The filters were blocked with 5% skim milk in Tris Buffered Saline with Tween-20 (TBS-T) (150 mM NaCl, 20 mM Tris-HCl (pH 7.6), 0.1% Tween-20) for 100 min and incubated with primary antibodies (diluted 1:1000 to 1:2000 with 5% skim milk in TBS-T) for 1 h at room temperature. The filters were then washed, incubated for 1 h at room temperature with the secondary antibody (sheep anti-mouse or donkey anti-rabbit) conjugated with horseradish peroxidase (Amersham Biosciences, Piscataway, NJ), and washed with TBS-T. Immunoblotted bands were detected by using the ECL system (Amersham Biosciences, Piscataway, NJ) with the same exposure time for all uses of a particular antibody.

## Results

### Model calibration; the first round of parameter fitting to experimental data

We performed model calibration with the generalized least squares method using multi-start solver, which mimics Monte-Carlo sampling of the initial parameter guesses.

For this study, we used the protein amount of cyclin D and cyclin E at each phase in the cell cycle. Additionally, we used the protein amount of total Rb (Supplemental Figure 1). The parameter fitting was performed for 12 parameters of 3 reaction species (cyclin D, cyclin E, and total Rb in nuclei; equals the sum of hypo- and hyper-phosphorylated Rb).

We chose part of the parameters for optimization because (1) in Yao's original paper, they indicated that a part of the model parameters comes from experiments, so we decided to keep the original values, and (2) the other 12 parameters were estimated via numerical tests. We used these parameters for the fitting to our experimental data. And (3), the aim of using only a part of the parameters for fitting was to reduce error in the process of parameter estimation.

**Figure 2:**
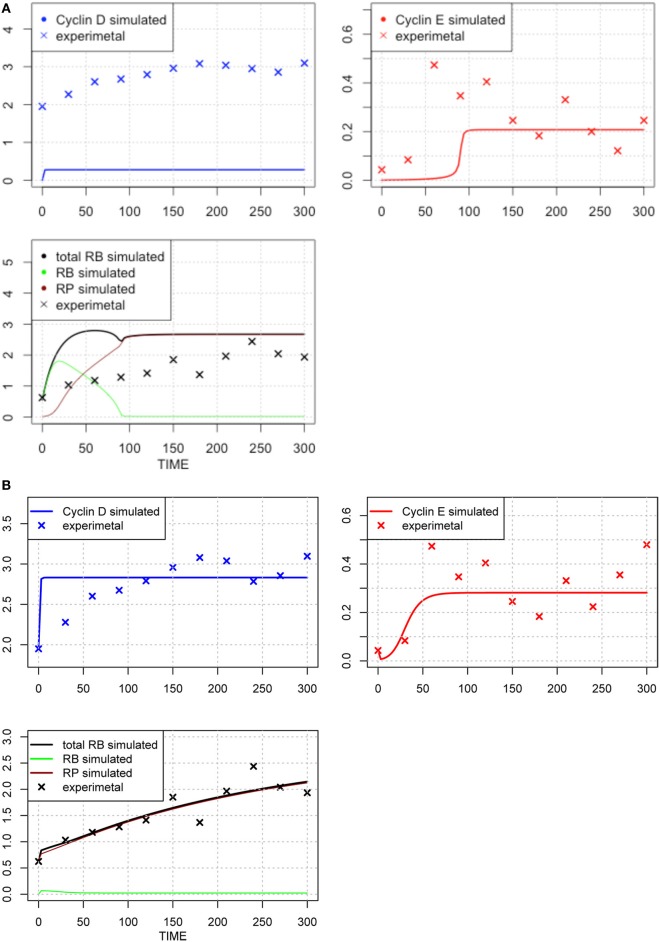
**Time-course of concentrations of proteins in the model**. The x-axis indicates the Time [min], the y-axis indicates the concentration of the species [nM]. The upper left panel shows Cyclin D (line: simulation result, cross: experimental result), upper right panel shows Cyclin E (line: simulation result, cross: experimental result), lower panel shows phosphorylated (brown line), dephosphorylated (green line) and their sum (black line) of simulation data, with experimental result (black cross). The three species were fitted to the experimental data. Simulation results were produced with the default set **(A)** and the 1st set of optimized parameter values **(B)**. Parameters chosen for optimization were those, which have not been estimated experimentally, so that the resulting simulation fits the qualitative behavior of the system. The parameters are: “kRE,” “kkE,” “kkM,” “kCDS,” “kR,” “KS,” “kkCE,” “KE,” “KCE,” “degRP,” “kkCD,” and “kb.” Optimized parameters are shown in Table 1, right-most column. The objective value for the fit in **(B)** is 1.18.

The original parameter set is shown in Table 1, middle column, and the results of optimization of the parameter values are shown in Table 1, right-most column. The time-course of each molecule with the original (A) and new parameter sets after the first round of parameter fitting (B) are shown in Figure 2. The optimized parameter produced closer curves to experimental data than the simulation results with the original parameter set. Now we performed local and global sensitivity analyses to test if these 12 parameters changed the sensitivity of the model to estimate how this parameter fitting affected the sensitivity of the model.

**Table 1:** **The original and 1st sets after parameter optimization**.

**Parameter names**	**Original parameter values**	**1st set of optimized parameter values**
dMC	0.70	−
dE	0.25	−
dCD	1.5	−
dCE	1.5	−
dR	0.06	−
dRE	0.03	−
kP1	18	−
kP2	18	−
kDP	3.6	−
KM	0.15	−
KCD	0.92	−
KRP	0.01	−
kRE	180	8.1647
kkE	0.4	19.977
kkM	1	0.081606
kCDS	0.45	4.9113
kR	0.18	0.013629
KS	0.50	0.53629
kkCE	0.35	1.1414
KE	0.15	19.996
KCE	0.92	18.890
dRP	0.06	0.0039885
kkCD	0.03	0.20762
kb	0.003	0.0000090144

*A minus sign means the same value as the original*.

### Local sensitivity analysis (LSA)

**Figure 3:**
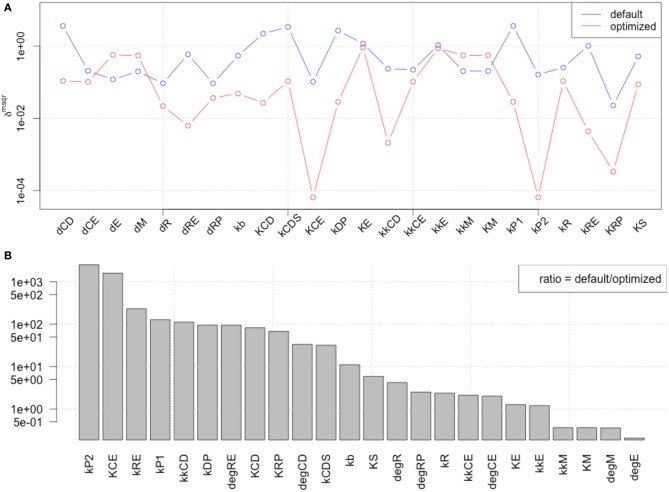
**Comparison of local parameter rank of the original parameter set with the 1st set of optimized parameters**. **(A)** The graph shows the rd_msqr_ of the original and 1st set of 24 optimized parameters. The blue line indicates the result of local sensitivity analysis with the original parameter set, and the red line indicates the result with the optimized parameter set. **(B)** Visualization of the changes in local sensitivity with the original and the 1st set of optimized parameters for the model. The ratio of each parameter sensitivity is indicated. The largest changes happened with parameters related to Rb protein metabolism, which is an inhibitor of the transcription factor E2F, or the metabolism of the transcription factors themselves, c-Myc and E2F, except kCE (the parameter relating to cyclin E concentration).

We performed LSA with published parameter values (Figure 3A, blue line) and with the 1st set of optimized parameters (Figure 3A, red line), and calculated the ratio between default and optimized in order to visualize the changes in local sensitivity of parameters (Figure 3B). LSA was performed for all 24 parameters in the model. The sensitivity analyses showed that the parameter optimization of the time-course data induced less sensitivity except for the parameters related to metabolism of transcription factors c-Myc (dM, kM, and kkM) and E2F (dE).

### Global sensitivity analysis (GSA) of observables

**Figure 4:**
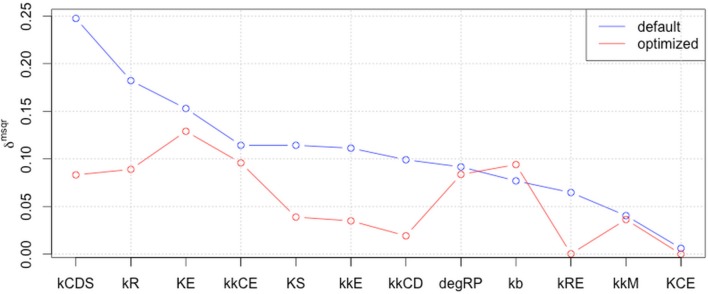
**Comparison of global parameter rank of the original parameter set with the 1st optimized parameter set**. The blue line indicates the result of local sensitivity analysis with the original parameter set, and the red line indicates the result with the 1st optimized parameter set. (optimized → opmitized).

Next, we performed GSA with the original and optimized parameters. We compared the sensitivities of 12 identified parameters and newly optimized parameters (Figure 4).

The result showed that the optimized parameters were less sensitive, except for one parameter related to c-Myc activity. These two kinds of parameter sensitivity analyses suggested a specific role for the transcription factors compared with the other reaction species in the model, the cyclins.

Next, we performed bifurcation analyses with the original parameter set and the 1st optimization parameter set to investigate the effect of parameter fitting to the model behaviors.

### First bifurcation analysis

**Figure 5:**
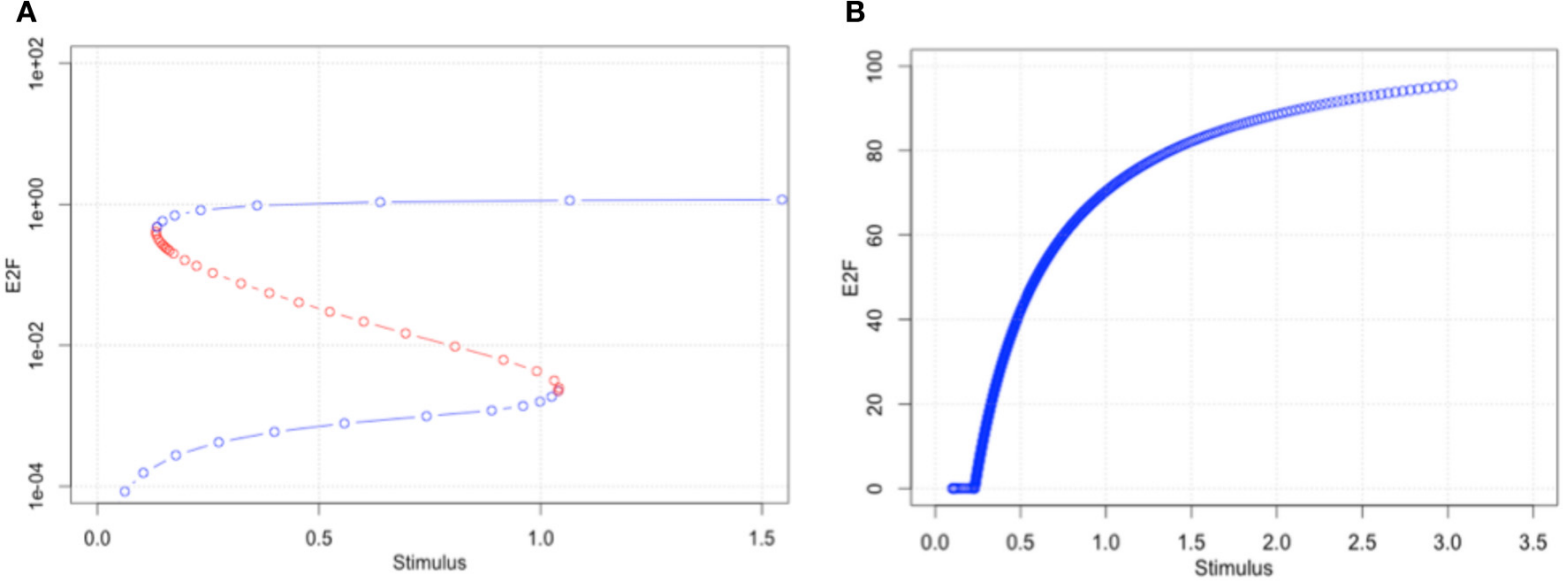
**The first bifurcation analyses with original parameter set (A) and optimized parameter set (B)**. The original parameter set produced bimodality and showed a wide range of hysteresis. By optimizing parameters to a malignant cell condition, the model has lost bimodality **(B)**.

We performed bifurcation analyses to investigate how parameter optimization using time-course data changed the dynamical characteristics of the model. The result with the original parameter set showed two bifurcation points, the so-called saddle nodes where the stable and unstable (blue and red, respectively) meet (Figure 5A). Bistability and hysteresis can be recognized in the model behaviors. On the other hand, the 1st set of optimized parameters showed transcritical bifurcation, i.e., a stable steady state becomes unstable and vice versa (Figure 5B). This means that the bimodality had been lost after the parameter optimization. This result further suggests that the key molecules to overcome the R-point, which are components of the model, seem to accumulate in the cell, and theoretically, cells that can no longer stop the accumulation by optimizing the parameter values convert to a malignant condition. Even if such conditions could actually be induced in a malignant cell, the cell line we used maintains contact inhibition and does not proliferate in an anchorage-independent manner.

Next, we performed a second parameter optimization by reconsidering the optimization target based on the results of our own sensitivity analyses and knowledge about *in vivo* biochemical reactions, and examined whether the newly optimized parameter set would rescue the model bimodality.

New biochemical insights were found by Aoki et al. (2011), where they showed that in the *in vivo* phosphorylation process, a target molecule that has two possible phosphorylation residues must have a different phosphorylation process than that *in vitro*. Based on this knowledge, we selected the parameters kP1 and kP2, which relate to Rb phosphorylation. At the same time, we excluded 4 parameters (KCE, kkCD, kRE, and KS) because of their low sensitivities in the results of both LSA and GSA. We aimed by this exclusion to produce a parameter set that had less sensitivity.

### New rounds of parameter optimization and sensitivity analyses

**Figure 6:**
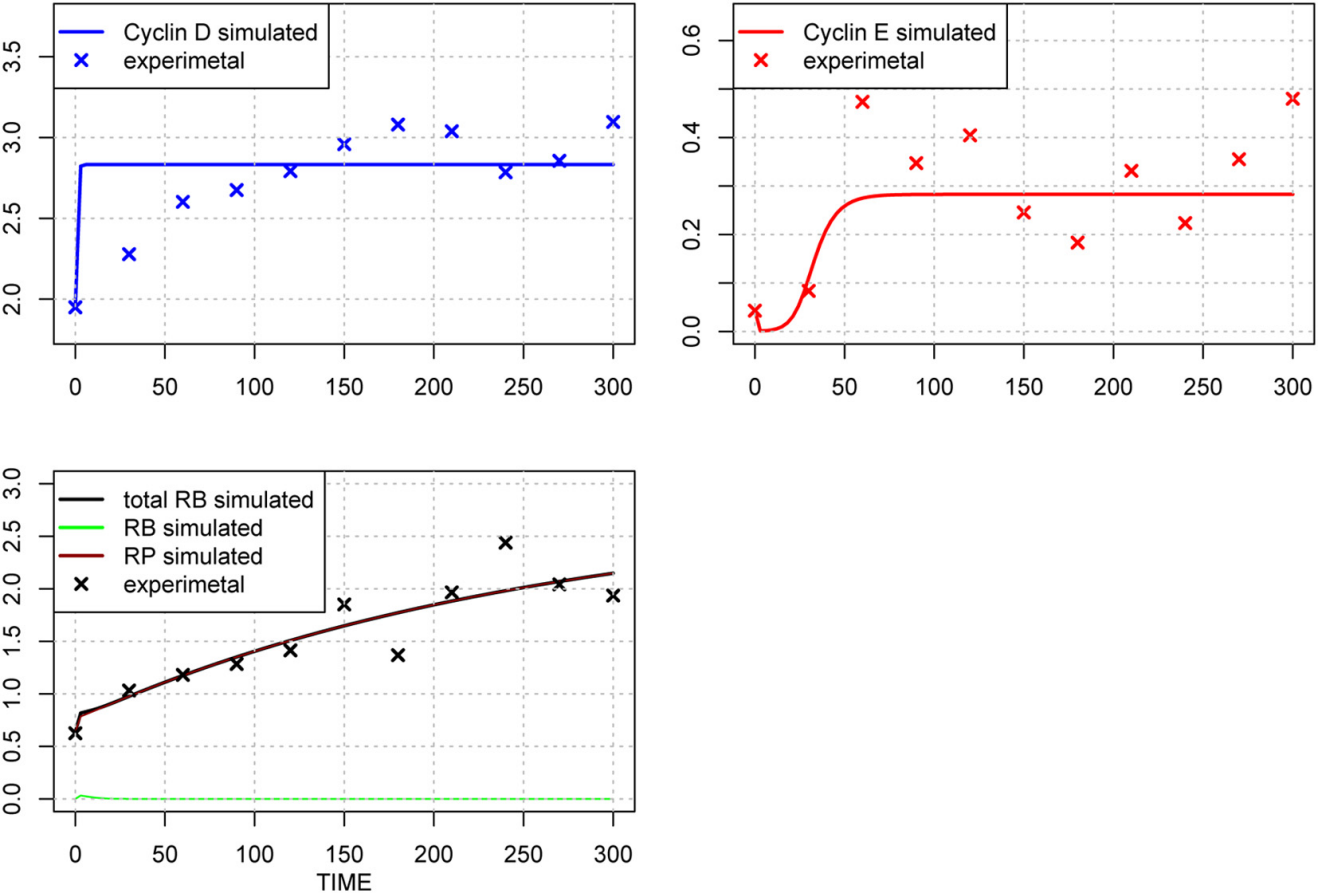
**Time-course of protein concentrations in the model**. The x-axis indicates the Time [min], the y-axis indicates the concentration of the species [nM]. The upper left panel shows Cyclin D (line: simulation result, cross: experimental result), upper right panel shows Cyclin E (line: simulation result, cross: experimental result), lower panel shows phosphorylated (brown line), dephosphorylated (green line) and their sum (black line) of simulation results, with experimental result (black cross). The fitting to the experimental data was repeated for the three species after the 1st set of parameter optimization.

**Figure 7:**
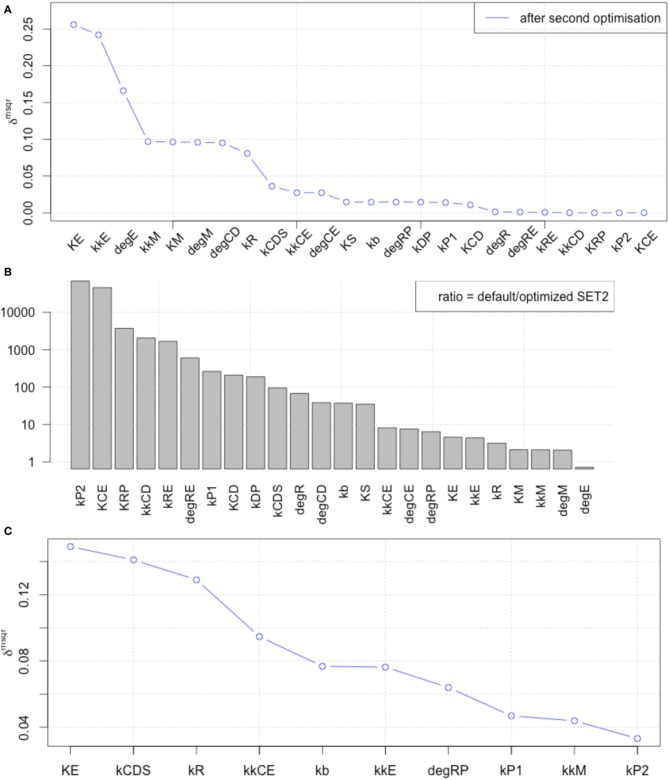
**The results of LSA (A,B), and GSA (C) with the 2nd set of optimized parameters**. All the parameters show less sensitivity than the original or the 1st set of optimized parameters.

We included the results of sensitivity analyses and performed a 2nd parameter optimization. The optimized parameters are indicated in Table 2, and the fitting results are shown in Figure 6. We performed sensitivity analyses with these 2nd sets of optimization parameters (Figure 7). Both LSA and GSA showed less sensitivity in total than the 1st set of optimized parameters. We used this 2nd set of optimized parameters for further bifurcation analyses.

**Table 2:** **Optimization results**.

**Parameter names**	**2nd optimization results of parameter values**
kkE	7.9962E + 01
kkM	1.0234E + 00
kCDS	4.9260E + 00
kR	1.2490E − 02
kkCE	9.3132E − 01
KE	1.5554E + 02
degRP	3.8691E − 03
kb	1.3559E − 05
kP1	2.1629E + 01
kP2	4.4433E − 02

*Newly optimized 1: normal bounds; newly optimized 2: the values are those estimated with smaller bounds (increasingly enlarged where necessary) used for estimation; newly optimized 3: “Km” included*.

### Second bifurcation analysis

**Figure 8:**
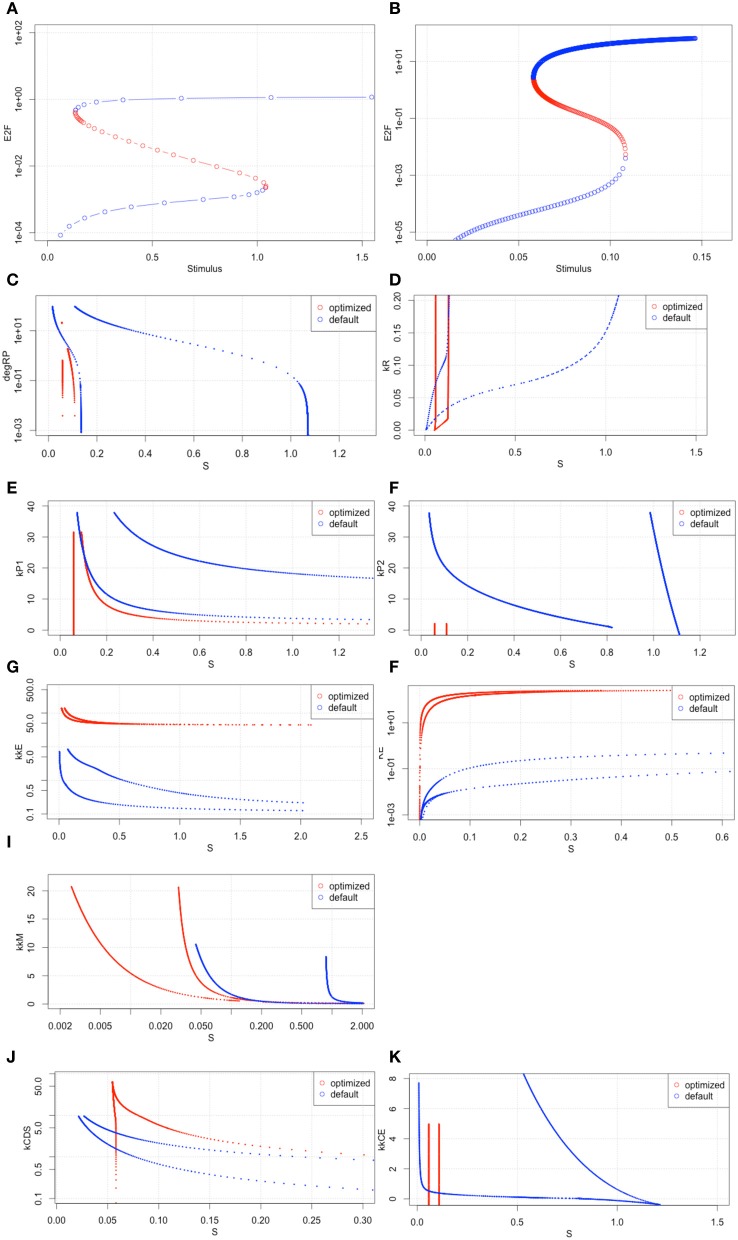
**Bifurcation analyses with the 2nd set of optimized parameters**. **(A,B)** the results of bifurcation analysis with the original parameter set and the 2nd optimized set. **(C–K)** Two parameter space analyses. All x-axes indicate *S* values. The y-axis of each graph indicates **(C)** degRP.

We performed a second bifurcation analysis with the newly optimized set of parameters (Figure 8). The 2nd set of optimized parameters showed bistability with a narrower range of hysteresis (Figure 8B). This result suggests that the sensitivity changed less than the original, but the model behavior changed to be more sensitive to the change of the extracellular stimulus level (S).

To investigate the bistable properties of the optimized model in more detail, we performed a two-parameter space analysis (Figures 8C–K). These results showed that the Rb and c-Myc active-inactive state changes could happen with relatively small amounts of extracellular stimuli (Figures 8C,E,I). These changes may affect the behavior of the two key cyclins, cyclin D and cyclin E. CyclinD is independent from the activity of E2F, and cyclin E is dependent on the activity of E2F. Cyclin D is required in an earlier stage of the cell cycle than cyclin E. Together, these results suggest that by fitting the model to a malignant cell, the model behaves such that cyclin D levels can easily accumulate with a small amount of extracellular stimuli, but once cyclin E starts to accumulate, there is no mechanism to stop the cell cycle. This could mean that the R-point does not work properly in the cell line we used.

This raises the question as to why the model behaved more sensitively after parameter optimization of the growth factor stimuli than in the original condition. Nevertheless, the parameters were optimized into less sensitive conditions. We designed and performed another parameter optimization to check if this alternation of model behavior was correlated with the sensitivity.

### Bistability independent of global sensitivity

**Figure 9:**
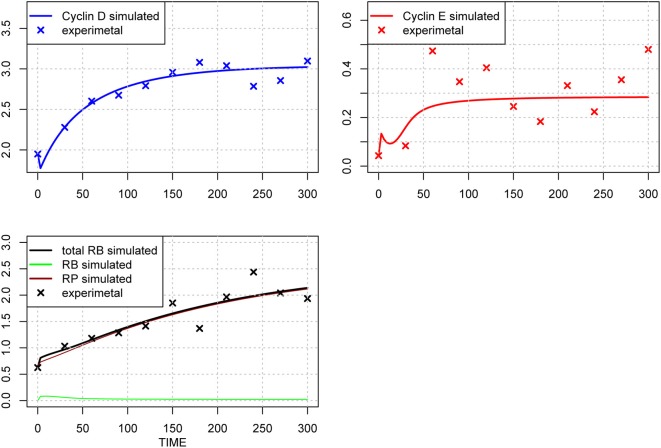
**Time-course of protein concentrations in the model**. The fitting to the experimental data was performed for the 3 species. The x-axis indicates the Time [min], the y-axis indicates the concentration of the species [nM]. The upper left panel shows Cyclin D (line: simulation result, cross: experimental result), upper right panel shows Cyclin E (line: simulation result, cross: experimental result), lower panel shows phosphorylated (brown line), dephosphorylated (green line) and their sum (black line) of the simulation results with experimental result (black cross). The objective value has decreased for this estimation round and is equal 0.67, which is visible in the improved fit of cyclin D.

**Figure 10:**
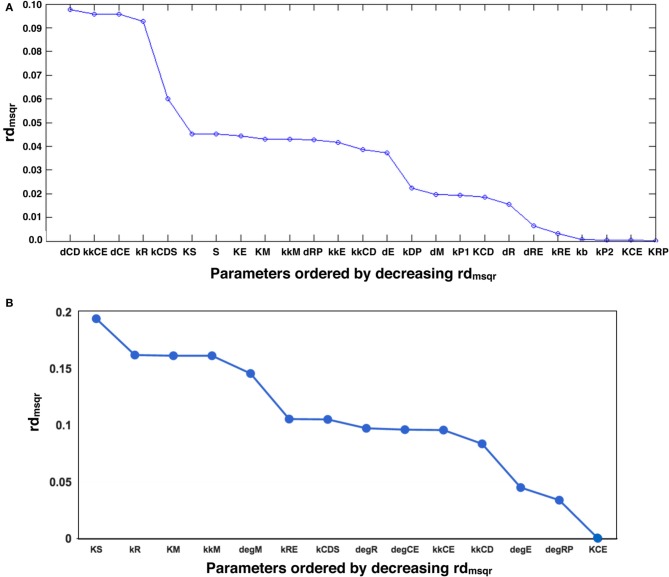
**LSA and GSA results of the third optimization of parameters**. **(A)** The results of LSA. There was no parameter that was more sensitive than the original. **(B)** The results of GSA. KS, kkM, and kRE were larger than the original parameter cases; and KS, KM, kkM, kRE, kCDS, kkCD, and kCE were larger than the 1st set of optimized parameters. At the same time, the following parameters were less sensitive than the original: kR, kCDS, kkCE, kkCD, dRP, and KCE, or than the first set of optimized parameters (dRP only).

We performed another parameter optimization in order to address parameter sensitivity and whether the bimodality of this model has causality. We optimized low sensitive parameters based on the sensitivity analyses results of the original parameter set (Supplemental Figure 3; dM, KM, kkM, dE, kRE, kR, dR, degRP, dCE, KCE, kkCE, kkCD, kCDS, KS). Figure 9 shows the time-course of 3 fitted species, and the optimized parameter values are listed in Table 3. The third optimization process allowed to make objective value smaller than the first round result (objective value of the first round parameter fitting: 1.18; objective value of the third round parameter fitting: 0.67). Even the fitting of siumulated curves to the experimental data were improved, the results of LSA indicated that we could not increase sensitivity at any parameter among the 24 (Figure 10A). On the other hand, GSA results showed that some parameters are more sensitive compared to the original parameters (3 parameters among 9 comparable parameters), and the 1st set of optimized parameters (7 parameters among 8 parameters) (Figure 10B). We performed bifurcation analyses with this parameter set; however, we did not see bistability of this model with the third set of optimized parameters. This result suggests that model bistability does not depend on the global sensitivity of parameters.

**Table 3:** **Results of the third optimization**.

**Parameter names**	**3rd optimization results of parameter values**
kRE	135.66
kCDS	4.9954
kR	0.015991
KS	2.8508
kkCE	1.6526
KCE	3.8474
kkCD	4.8351
KM	0.99638
kkM	0.017103
degM	0.01248
degE	0.18258
degR	0.071878
degCE	4.8474
degRP	0.005211

## Discussion

We showed our results of model fitting to sparse time-course data. Generally, even if the data can cover only some of the variables, parameter optimization can change the model behavior to be different than the original. In our case, the original model indicated a healthy proliferating mechanism in that case R-point should work strictly. On the other hand, cancer cells are believed not to have proper R-point mechanisms; as a result, a cell can overcome the R-point with a small amount of growth factors. Our results show that at least some cancer cell-like properties can be produced via parameter optimization to time-course data of malignant cell lines (Figure 8).

We tested if the bistability of the model is correlated with the sensitivity of the parameters, because we aimed to reduce the parameter sensitivities by optimization to make the model behavior robust against parameter changes; however, the range of hysteresis had been reduced via parameter optimization, and as a result, the bistability of the model became unstable with a small change of extracellular stimuli (S, Figure 8). Our results did not suggest that the bistability of this model is dependent on the parameter sensitivity. Moreover, our results, which suggest the significance of the transcription factors and different behaviors of cyclin D and cyclin E, may indicate that the bistability of the cell cycle machinery could depend more on the strict context of the activation processes of these molecules.

The choices of the parameters for the second optimization were based on the results and the hypothesis by Aoki et al. (2011). Our idea is if we accept their hypothesis, the reason why *in vivo* specific double phosphorylation process happens is intracellular crowding. And it is independent from the specific molecular binding such as anchorage protein for MAPK. Then, the hypothesis should stand generally for *in vivo* double phosphorylation of single substrate. Therefore, we re-optimized the parameters of double phosphorylation processes of Rb. On the other hand, Rb protein has many other phosphorylation sites (Rubin et al., 1998). More than 10 phosphorylation sites of this protein had been counted. The parameter values may be different for each reaction of phosphorylation. However, we possibly estimate the difference would not affect to the critical behaviors of the model, such as bistability, etc. Because we have assumed that the multi-phosphorylation step of single substrate is a linear system, instead of a system, which shows switch-like, non-linear behavior, based on the results of Aoki's 2012 (Aoki et al., 2011). In this case, we may contract these multiple reactions into shorter steps as follows. When the first phosphorylation step shows linear process, and double phosphorylation also, and further, too, these reaction schemes are characteristically the same with a signal cascade which simply activates the next reaction species sequentially. We may describe this type of signal cascade with the first species and the last species with single activation reaction. Multiple-phosphorylation case is the same the sequentially activating cascade if the systems is essentially linear. We may describe the whole reaction process with the first site and the last site, and it seems double-phosphorylation reaction. We cannot eliminate the possibility that the reaction step includes actually multi-phosphorylaitons over two, then the parameter value may be multiplied into some other value. However, the change will not make strong impact to the bistable behavior of the entire model.

There could be another reason why the model property changes via parameter optimization, which is a more specific condition. One possible reason for the change of bifurcation behavior and its consequences is the difference of cell synchronization method of the fitting materials. By comparison with Yao's Supplemental Figure 2, however, the synchronization level of our sample seems the same or better than that of their cells (Supplemental Figure 1); therefore, this may not be the reason for that weak bistability is produced. This means that we may not simply conclude that the cellular synchronization condition affected the behavior of the optimized model. On the other hand, the timing of synchronization seems different between Yao's experimental data and ours, and this could affect the bistable property. The cells we used showed quicker cell cycle than the case of Yao's experiments. This is consistent with the results of bifurcation analyses, which showed the smaller jump and hysteresis from a state to the other, which means overcoming cell cycle checkpoint, in this case R-point, and moving to the next phase, in the words of cell cycle. The loose restriction at R-point could results short cycle of cellular proliferation.

We had found there exist three different types of parameter conditions in the correlation with the model bistability; one is the original (default) condition by Yao's work. The condition produces clear bistability. The second condition is the 2nd round parameter set in this paper or the parameter set for Supplemental Figure 4, which can produce narrow range of bistability. The last is which produced the best fitting results to our time-course data of Cyclin D (3rd round of this paper) or E (Supplemental Figure 5), however the both of these parameter sets could not produce bistability. Among our limited results, the following 4 parameters showed straightforward trends as the condition to reproduce bistability of the model. kRE contributes bistability when it takes only the value 180 ~ 194, both less or larger than it cannot produce bistability. As same as the case of kRE, kCDS can take less value than 4.926, kkCE can take less value than 1.1414, KCE can take less value than 1.0793 to reproduce bistability of the model. These parameters affect almost all of the time course of molecular concentration except c-Myc ([MC]). This may happen according to the characteristics of our material cells, Rat fibroblast 3Y1. This cell line does not express c-Myc before receiving the depletion signal of growth factor in culturing medium (Tsuneoka et al., 2003). We need to investigate both the theoretical properties of the model and biological data to make them consistent with each other.

These results indicate that even sparse and noisy experimental data can be used to improve a mathematical model by fitting to those data. In the case of Yao's model and our experiments, the parameter optimization allowed the model to adapt to physiological (cancer cell) conditions, even though the experimental data did not include enough information to identify the whole the parameter set, but instead suggested one relevant set of parameters to reduce the sensitivity against changes and to maintain bistability.

When we need to identify the whole parameter set, we should add more experimental data for other molecules, or perform more optimization with a different set of initial conditions. Partial evidence for the potential of changing initial conditions was shown in our several rounds of parameter optimization (Figures 2, 7, 9). We could produce better fitting to the experimental data by performing several rounds of parameter optimization; however, at the same time, the new parameter set changed the model behavior fundamentally (Figures 5, 8), and the possible causes may involve changes in the dynamics of molecules that lack experimental evidence. This means that providing experimental data for those molecules which have not yet provided experimental data for fitting would improve parameter optimization.

In this study, we did not perform practical identifiability analysis to consider if the model unknowns may be uniquely estimated under given experimental conditions. The results from practical identifiability may helpful to assess parameter estimate reliability and to compare possible experimental designs. Such analysis is especially important to improve experimental design. To perform this analysis, we need to be careful with noise. Fortunately, however, a lack of practical identifiability is not critical for its solvability. Adequate global optimization solvers can be employed to deal with the presence of suboptimal solutions.

In total, our results showed that optimizing parameters by using experimental data is useful to get the model closer to physiological conditions, even if experiments have not yet fully shown the effect on the targeting system. At the same time, we need enough resolution from experiments to provide good identifiability for the model parameters.

In the future, we will perform Optimal Experimental Design (OED) to determine a dynamic scheme of the measurements that generates the richest information in order to estimate parameters with greater precision. To provide measurements that maximize the quantity and quality of the information provided by the experiments while minimizing the experimental burden is the desired goal to connect practical experimental information with mathematical models of molecular mechanisms.

### Conflict of interest statement

The authors declare that the research was conducted in the absence of any commercial or financial relationships that could be construed as a potential conflict of interest.

## Appendix

### Ode equations

The following is the full ODE system for the Yao 2008 model. S stands for the systems' forcing function, in the form of the serum concentrations, which here is a constant with values for the whole duration of the experiment/simulation of 0.5 and 3%.

$$\begin{array}{l} \frac{d\left[MC\right]}{dt}=\frac{kkM\left[S\right]}{KS+\left[S\right]}-dMC\left[MC\right]\\ \;\frac{d[EF]}{dt}=\frac{kkE\left[MC\right][EF]}{\left(KM+\left[MC\right])(KE+\left[EF\right]\right)}+kb\frac{\left[MC\right]}{KM+[MC]}-dE\left[EF\right]-kRE\left[RB\right]\left[EF\right]+\frac{kP1\left[CD\right]\left[RE\right]}{KCD+\left[RE\right]}+\frac{kP2\left[CE\right][RE]}{KCE+\left[RE\right]}\\ \;\frac{d[CD]}{dt}=\frac{kkCD[MC]}{KM+[MC]}+\frac{kCD[S]}{KS+[S]}-dCD[CD]\\ \;\frac{d[CE]}{dt}=\frac{kkCE[EF]}{KE+[EF]}-dCE[CE]\\ \;\frac{d[RB]}{dt}=kR+\frac{kDP[RP]}{KRP+[RP]}-kRE\left[RB\right]\left[EF\right]-\frac{kP1\left[CD\right]\left[RB\right]}{KCD+\left[RB\right]}+\frac{kP2\left[CE\right]\left[RB\right]}{KCE+\left[RB\right]}-dR[RB]\\ \;\frac{d[RP]}{dt}=\frac{kP1\left[CD\right][RB]}{KCD+[RB]}+\frac{kP2\left[CE\right][RB]}{KCE+[RB]}-\frac{kDP\left[RP\right]}{KRP+\left[RP\right]}+\frac{kP1\left[CD\right]\left[RE\right]}{KCD+\left[RE\right]}+\frac{kP2\left[CE\right]\left[RE\right]}{KCE+\left[RE\right]}-dRP[RP]\\ \;\frac{d[RE]}{dt}=kRE\left[RB\right]\left[EF\right]-\frac{kP1\left[CD\right]\left[RE\right]}{KCD+\left[RE\right]}-\frac{kP2\left[CE\right]\left[RE\right]}{KCE+\left[RE\right]}-dRE[RE] \end{array}$$
