# Supplementary material for: Assessing uncertainty in model parameters based on sparse and noisy experimental data
Source: Front Physiol. 2014 Apr 4;5:128. doi: 10.3389/fphys.2014.00128 (PMC3983526; doi:10.3389/fphys.2014.00128)
Supplement: Supplementary file 1 [file DataSheet1.ZIP › 68734_Hiroi_Supplementary_Files.pdf]

Assessing uncertainty in model parameters based on sparse and noisy experimental data

Authors: Noriko Hiroi*[$], Maciej Swat* and Akira Funahashi

* NH and MS are equal contributors
[$] Corresponding author hiroi@bio.keio.ac.jp

Supplementary Figures

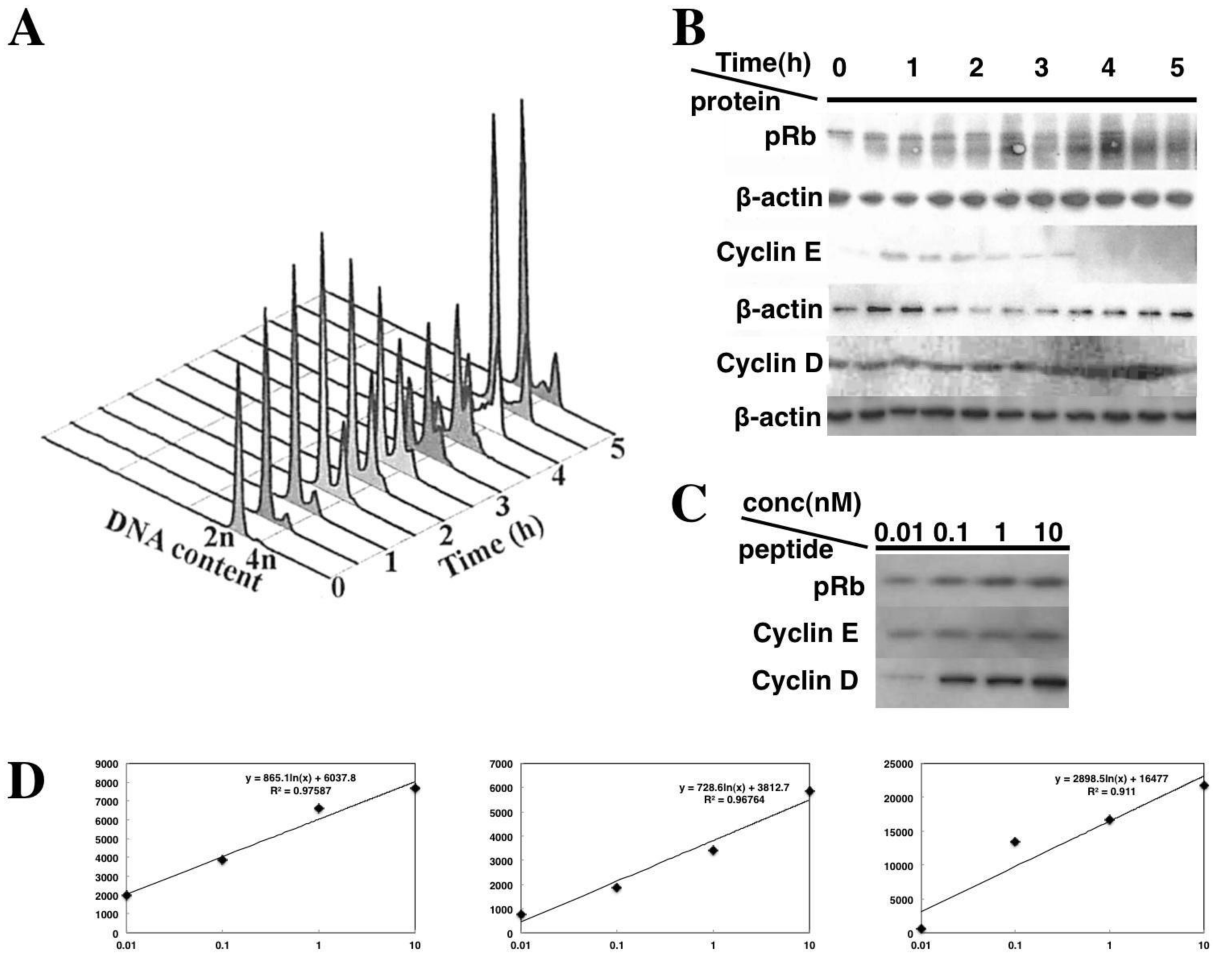

Supplemental Figure 1. Time-course analysis of DNA content of cells (A) and western blot results of target proteins (B). A. DNA content of cell populations after the release from thymidine. The cells that contain 2n DNA should be arrested in G1 to S phase. The cells in S and early G2 phase show 2 to 4n DNA. The cells in late G2 to middle M phase appear as 4n. B. Western blot analyses of target proteins during the cell cycle. (C) Western blot results of each peptide. We calculated the calibration curves (D) of pRb (left), cyclin E (middle), and cyclin D (right) from the results in (C). The x-axes represent the concentration of each protein [nM], the y-axes represent the relative intensities of bands detected on the membrane with X-ray film and calculated by ImageJ.

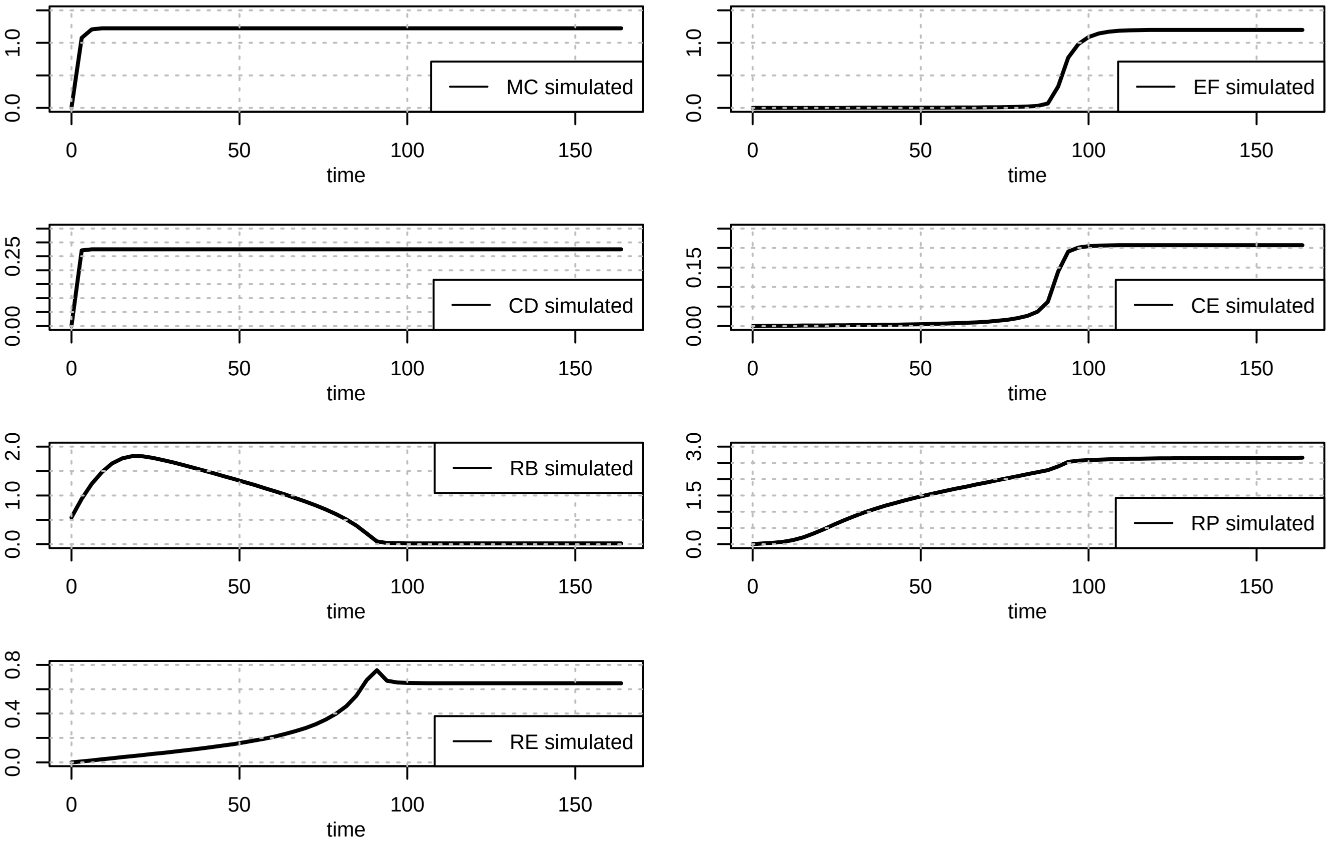

Supplemental Figure 2. The original time-course results of Yao's cell cycle model. We confirmed that our reconstructed model can produce the same results as the original paper.

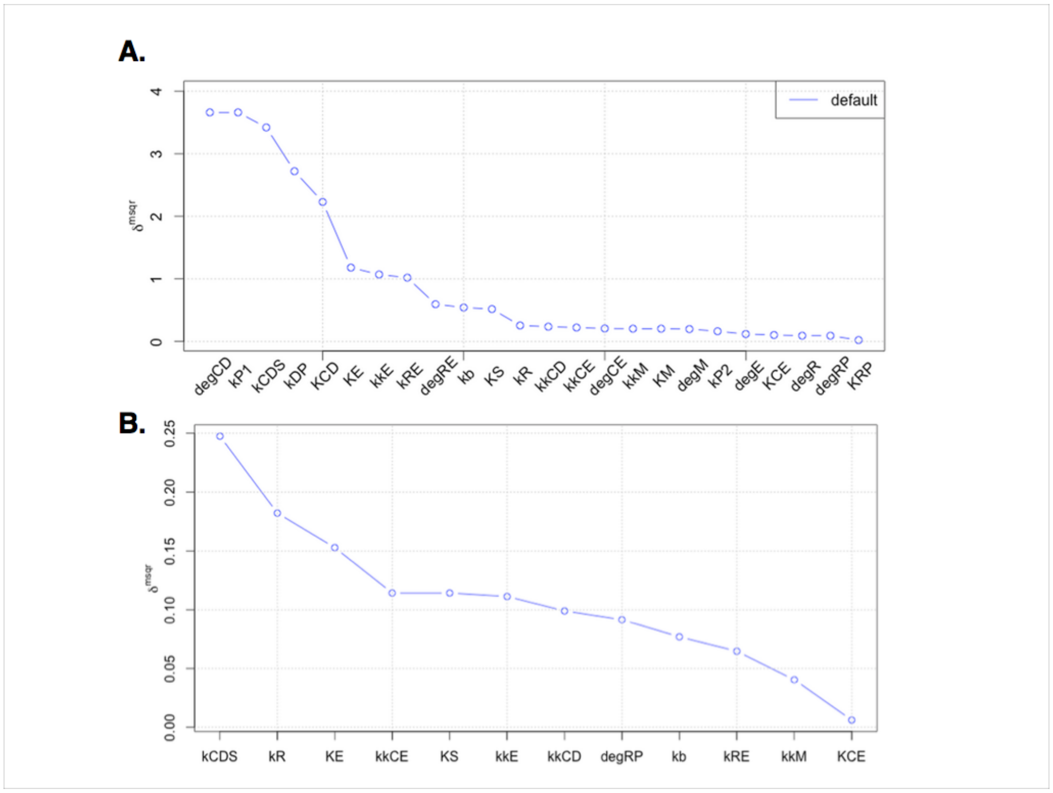

Supplemental Figure 3. LSA (A) and GSA (B) results of the original parameter set.

A. Fitted variables to experimental data

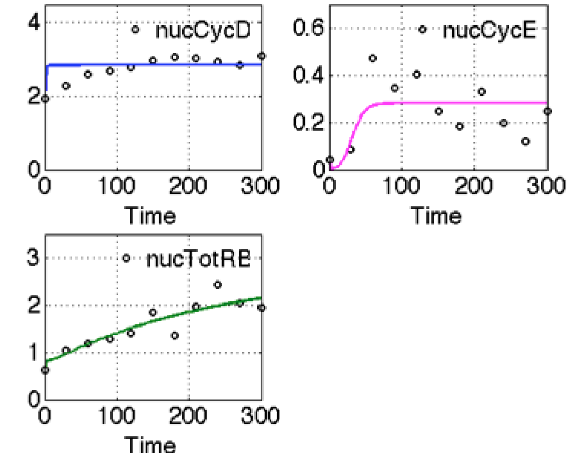

B. Plots with all species with new parameter set

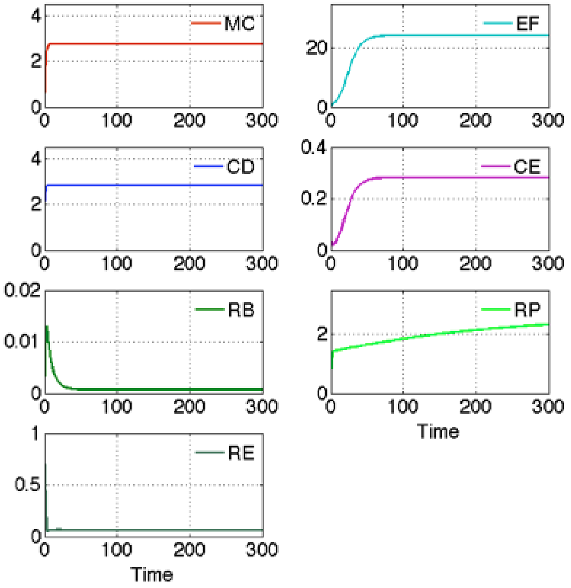

C. The result of bifurcation analysis with optimized parameter values

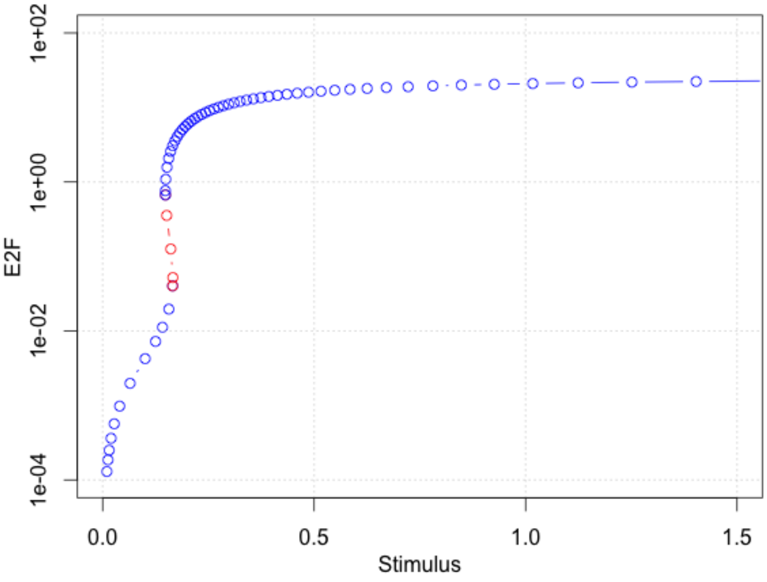

Supplementary Figure 4

A. Fitted variables to experimental data

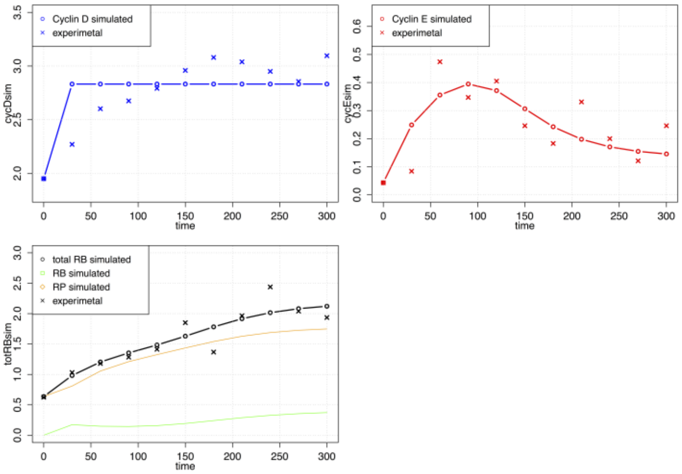

B. Plots of all other species with new parameter set

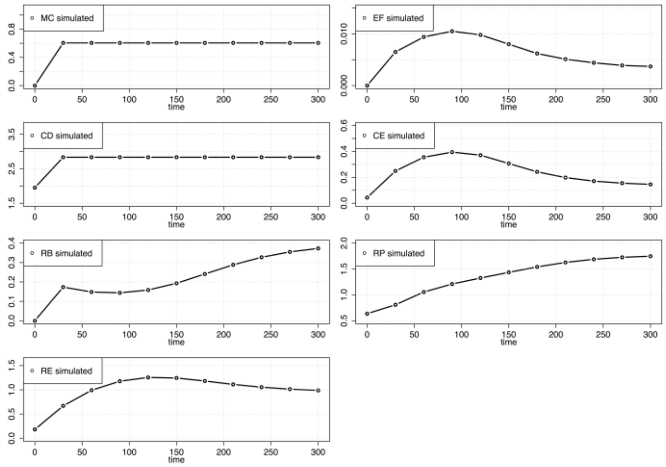

C. The result of bifurcation analysis with optimized parameter values

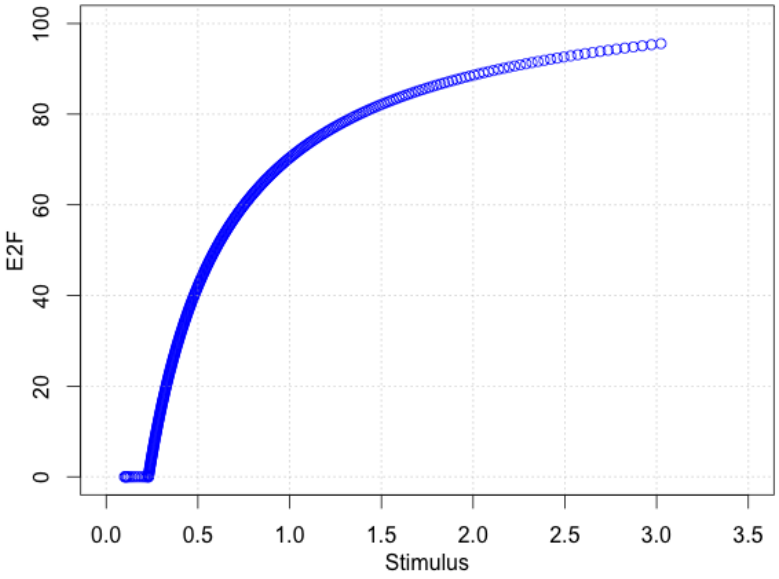

Supplementary Figure 5

Supplementary Tables

Supplemental Table 1. The parameter values

PARAMETER	Initial value	Optimized values
kRE	180	194.1400
kEF_prod	0.4	16.3900
kM	1	4.6234
kCDS	0.45	4.4140

kRB	0.18	0.0130
KS	0.5	4.1685
kCE	0.35	1.0793
KEF	0.15	37.7980
KE	0.92	1.0363
degRP	0.06	0.0040
kCD	0.03	2.5322
kb	0.003	0.0008
INITIAL CONDITIONS		
CycD	1.5	1.9503
CycE	0.5	0.0425
RP	0.5	0.6369

Supplemental Table 2 The parameter values

PARAMETER	Initial value	Optimized value
kRE	180	1.0200e+00
kEF_prod	0.4	9.9916e+01
kM	1	1.4324e-01
kCDS	0.45	4.9383e+00
kRB	0.18	1.3743e-02
KS	0.5	
kCE	0.35	9.5793e-01
KEF	0.15	1.1996e+02
KE	0.92	1.9494e+01
degRP	0.06	4.0545e-03
kCD	0.03	
kb	0.003	1.3384e-05
INITIAL CONDITIONS		
CycD	1.5	1.9503
CycE	0.5	0.0425

RP	0.5	0.6369

Supplementary Material

Supplemental Material 1. SBML file of Yao's 2008 model created with CellDesigner (Yao2008.xml).

Supplemental Material 2. Matlab file with the model encoded to be used with the AMIGO toolbox (Yao2008A_PE_paper.m).
